# Smartphone-based non-invasive biofeedback therapy for post-stroke sleep disorders: short report

**DOI:** 10.3389/fneur.2025.1601821

**Published:** 2025-06-25

**Authors:** Jisoo Park, Minyong Jung, Jiyeon Ha, Jonghwa Jeonglok Park, Sun Im

**Affiliations:** ^1^Department of Rehabilitation Medicine, Bucheon St. Mary’s Hospital, College of Medicine, The Catholic University of Korea, Seoul, Republic of Korea; ^2^BELL Therapeutics Inc, Seoul, Republic of Korea; ^3^Intelligence and Interaction Research Center, Korea Institute of Science and Technology, Seoul, Republic of Korea

**Keywords:** biofeedback, stroke, stroke rehabilitation, sleep initiation and maintenance disorders, insomnia, digital health, digital therapeutics

## Abstract

Post-stroke sleep disorders (PSSDs), particularly insomnia, are common yet insufficiently recognized complications that can negatively affect recovery in stroke patients. Existing treatment options are often hindered by side effects, complex protocols, or cumbersome equipment. This short report introduces a smartphone-based biofeedback intervention designed to address insomnia by targeting autonomic nervous system (ANS) dysfunction. The intervention was tested on three subacute stroke in-patients unresponsive to pharmacological therapies. All patients demonstrated significant improvements in subjective sleep quality, assessed primarily with the Korean version of the Richards-Campbell Sleep Questionnaire (RCSQ) and secondarily with the Insomnia Severity Index (ISI-K) and Pittsburgh Sleep Quality Index (PSQI-K). RCSQ results indicated improvements in subjective sleep quality across all patients. ISI scores showed all three patients achieved remission thresholds for insomnia, with improvements exceeding the Minimal Clinically Important Difference (MCID). PSQI-K scores also improved in all cases, with two patients reaching threshold levels for insomnia. The intervention’s efficacy was validated through an on–off–on design, as improvements were observed during intervention periods, diminished during washout phases, and resurfaced with reintroduction. Results highlight the potential of a fully non-invasive solution for managing insomnia in stroke patients, offering a practical and effective alternative to traditional treatments.

## 1 Introduction

Post-stroke sleep disorders (PSSD) affect up to 78% of stroke survivors and are significant yet insufficiently recognized complications (1). These disorders impede functional recovery and rehabilitation after stroke, as sleep promotes neurogenesis and neural rewiring process in damaged brains (2, 3). Indeed, insomnia in acute stroke patients is associated with poorer outcomes, reduced quality of life, and an increased risk of secondary strokes (4). Despite their high prevalence and serious consequences, PSSD remains insufficiently addressed in standardized care guidelines, highlighting a critical gap in stroke management (5).

Based on the diagnostic tools, including the Diagnostic and Statistical Manual of Mental Disorders, Fifth Edition (DSM-V) and Fourth Edition (DSM-IV) (6, 7), up to 32% of stroke patients are diagnosed with insomnia as part of PSSDs within the first few months of stroke onset, compared to a 6% prevalence in the general population (5, 8). This high prevalence rate denotes the need for early diagnosis and treatment of post-stroke insomnia in acute settings. However, existing treatments are primarily tailored to chronic insomnia and are unsuitable for the acute stroke context (9). For instance, Cognitive Behavioral Therapy for Insomnia (CBT-I), the gold standard for treating chronic insomnia, is effective when delivered in four to eight weekly sessions over a 4–12-week period and requires active participation in physiologically and mentally demanding techniques, including techniques such as stimulus control, sleep restriction, and cognitive restructuring (10). Although effective, this approach is not ideal for acute stroke patients due to its delayed onset of action and the high prevalence of stroke-related motor and cognitive impairments.

Pharmacotherapy is commonly used to manage insomnia due to its accessibility and rapid onset of action. Recommended drug classes for chronic insomnia include benzodiazepines, non-benzodiazepine hypnotics, and orexin receptor antagonists. However, acute insomnia in stroke patients remains largely unaddressed, with no specific pharmacological guidelines available (11, 12). Additionally, pharmacological options carry distinct risks for stroke patients. For instance, benzodiazepines, commonly prescribed for insomnia, are contraindicated because they can exacerbate breathing-related sleep disorders and motor deficits (8, 13). Other drug classes, while potentially usable, carry risks of oversedation, delirium, and hypotension, which can hinder rehabilitation and exacerbate stroke related impairments (14).

These limitations underscore the need for alternative interventions tailored to the unique challenges faced by stroke patients. Smartphone-based Digital Cognitive Behavioral Therapy (dCBT) offers a scalable and cost-effective solution with proven efficacy in managing chronic insomnia (15, 16). However, similar to traditional CBT, it requires extended sessions and cognitive engagement, typically involving six or more sessions over a minimum of 4 weeks to achieve significant results (17). Non-invasive brain stimulation techniques, such as Repetitive Transcranial Magnetic Stimulation (rTMS) and Transcranial Direct Current Stimulation (tDCS), have shown promise in enhancing sleep quality, yet they are constrained by the need for specialized equipment and operators, high costs, and the potential for discomfort and side effects (18–20).

Biofeedback therapy, which provides real-time feedback on physiological parameters such as breathing patterns and neural activity, offers a non-invasive approach to modulate the autonomic nervous system (ANS). This method has demonstrated effectiveness in improving sleep quality across diverse populations (21–24), yet it is limited by the requirement for bulky equipment, specialized expertise, and extensive patient training. Given these challenges, there is a pressing need for accessible and effective solutions tailored to stroke patients suffering from insomnia in acute care settings. A recent proof-of-concept study experimented with mobile-based bio-sonification therapy, a modified form of biofeedback, and found that its use in individuals with insomnia symptoms led to significant improvements in sleep onset and maintenance (25). This approach offers rapid relief while eliminating the need for specialized equipment, extensive patient training, and expert oversight, highlighting the potential of digital biofeedback therapy for insomnia treatment. This study aims to assess the feasibility and effectiveness of a smartphone-based biofeedback intervention for post-stroke insomnia refractory to conventional pharmacological treatments, using an on–off–on design to evaluate its impact on sleep quality (25).

## 2 Methods

### 2.1 Study protocol

The study population consisted of three inpatients without any prior history of stroke before the index event, all of whom subsequently developed insomnia. The inclusion criteria required that patients had not responded to standard pharmacotherapy for sleep disturbances. The intervention utilized a mobile application, which was applied twice daily.

**Figure 1:**
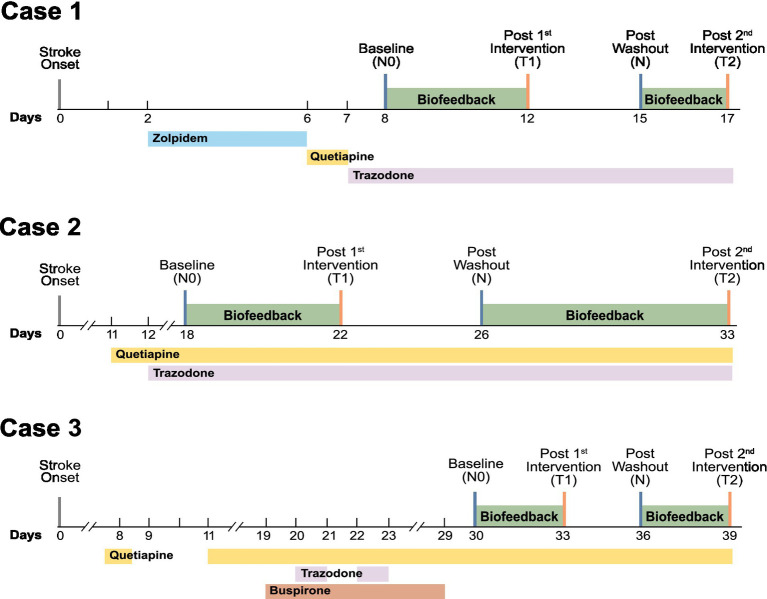
The timeline of pharmacological and application-based interventions for post-stroke insomnia includes four assessment time points: baseline (N0), completion of the first biofeedback intervention (T1), post-washout period (N), and completion of the second biofeedback intervention (T2).

A repeated on–off protocol was employed, consisting of three to seven consecutive nights of intervention followed by a three- to four-night washout period, after which the intervention was resumed. The exact duration of each phase varied slightly due to clinical and logistical constraints inherent in the inpatient setting, including therapy schedules and discharge planning, while ensuring sufficient exposure to observe treatment effects. This protocol was specifically selected because, despite sharing a diagnosis of PSSD, the patients differed substantially in their clinical characteristics, including infarcted brain regions, concurrent medications, and treatment timelines. By enabling direct within-subject comparisons of pre-treatment, washout, and post-treatment conditions, the repeated on–off design minimized inter-subject variability and enhanced the reliability of findings, thereby mitigating concerns arising from the limited number of participants in this study. Figure 1 provides a timeline-based summary of each patient’s pharmacological treatment history and the schedule of the application-based intervention.

The patient’s conditions were assessed through verbal interviews at four distinct time points: the start of the first intervention, which served as the baseline; the end of the first intervention; the start of the second intervention following a washout period; and the end of the second intervention. Safety monitoring was performed by caregivers and nursing staff during routine rounds. The study’s termination criteria included the occurrence of a critical medical condition requiring transfer to the intensive care unit (ICU) or a recurrence of stroke.

### 2.2 Biofeedback intervention

This study employed a smartphone-based biofeedback therapy (pipeline code: BELL-001) developed by BELL Therapeutics Inc. (Seoul, Republic of Korea). This software was developed in compliance with the ISO 13485 standard for medical device development, and its efficacy, safety, and usability were evaluated in the proof-of-concept study before being applied to our case (25). Minimum hardware requirements are provided in Supplementary file 1.

**Figure 2:**
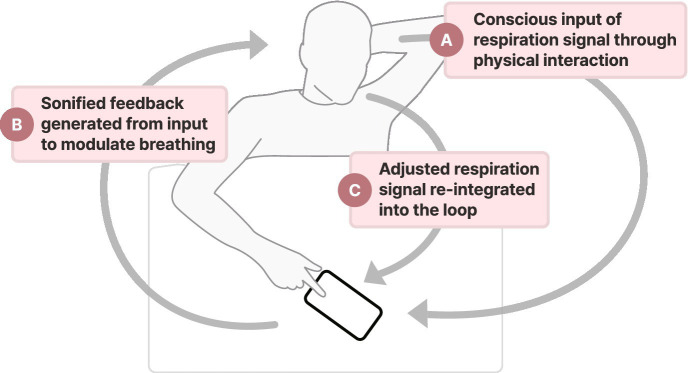
The biofeedback system consists of three primary components: **(A)** patient input of respiratory patterns by pressing and holding the screen during inhalation and releasing it during exhalation, **(B)** real-time sonified feedback of respiratory signals, and **(C)** modulation of breathing patterns guided by audio cues to promote slower and controlled breathing. This closed-loop feedback mechanism aims to facilitate autonomic regulation and enhance respiratory awareness.

To fit typical ward routines and support circadian stabilization, sessions were scheduled twice daily at 4:00 PM and 6:00 PM, with lights turned off at 8:00 PM. Patients were instructed to lie down in a comfortable position and launch the smartphone-based application. The intervention required them to manually press and hold the screen during inhalation and release it upon exhalation, allowing the application to capture breathing patterns and generate real-time auditory feedback to promote slower, deeper breathing. Once a stable respiratory signal was detected, users could transition to automated mode, wherein manual input was no longer required. In this mode, the system continued to deliver auditory cues to reinforce a sustained, regulated breathing rate. Each therapy session started with approximately 5 min of biofeedback intervention in manual input mode, followed by 25 min of automated mode (25). For individuals experiencing severe stroke-related impairments, the manual mode could be operated with caregiver assistance. Figure 2 provides a schematic representation of the biofeedback intervention.

### 2.3 Case 1

A 44-year-old male patient presented with sudden onset of right-sided weakness and was subsequently diagnosed with an infarction involving the left basal ganglia and periventricular white matter. After receiving initial conservative care in the stroke unit, the patient was transferred to the rehabilitation department for PSSD management. The patient was initially prescribed Zolpidem tartrate for insomnia, however, it failed to alleviate his sleep disturbances. Due to the lack of improvement, Zolpidem was discontinued, and Quetiapine fumarate was prescribed. However, this change resulted in dizziness as a side effect, further complicating the patient’s treatment. As an alternative, Trazodone hydrochloride was subsequently administered, but it also failed to enhance sleep induction. Given the persistence of his sleep issues despite pharmacological interventions, the biofeedback application was introduced as an adjunct therapy. The patient self-registered the initial respiratory data required by the application.

### 2.4 Case 2

A 58-year-old male patient presented with sudden left-sided weakness, which was subsequently diagnosed as a right middle cerebral artery territory infarction. The patient underwent mechanical thrombectomy followed by stent-assisted coil embolization. After postoperative care in the intensive care unit, he was transferred to the rehabilitation department to continue his recovery. Quetiapine fumarate was initially prescribed, but it did not resolve his sleep disturbances. Trazodone hydrochloride was added to the treatment regimen, yet sleep quality issues persisted. In light of these challenges, the biofeedback application was introduced as an adjunct therapy to complement the existing pharmacological treatments. Due to the patient’s impaired motor function, a caregiver assisted in manually inputting the respiratory patterns during the initial step.

### 2.5 Case 3

A 50-year-old male patient presented with sudden right-sided weakness due to a pontine intracerebral hemorrhage. After receiving conservative stroke care, the patient was transferred to the rehabilitation department for further management and recovery. The patient’s nocturnal agitation was managed with a combination of pharmacological interventions, including Buspirone hydrochloride, Trazodone hydrochloride, and Quetiapine fumarate. Despite these efforts, the patient continued to experience poor sleep quality, which significantly impacted his rehabilitation progress. Given such limitations, the biofeedback application was introduced as an adjunctive therapeutic strategy. Due to the patient’s impaired motor function, a caregiver assisted in manually inputting the respiratory patterns during the initial step.

### 2.6 Outcome measures

Subjective sleep quality was evaluated using three validated self-reported questionnaires. The primary outcome measure was the Korean version of Richards-Campbell Sleep Questionnaire (K-RCSQ), which assesses sleep depth, latency, awakenings, return to sleep, and overall sleep quality on a 0–100 scale, with higher scores indicating better sleep. RCSQ was selected as the primary outcome measure due to its validation as a reliable alternative to polysomnography in ICU patients (26). Secondary outcomes included the Korean versions of Pittsburgh Sleep Quality Index (PSQI-K) and the Insomnia Severity Index (ISI-K), both of which are recognized for their high sensitivity in evaluating sleep disturbances in stroke populations (27, 28). The score thresholds for diagnosing insomnia in both questionnaires were defined based on previous validation studies (29, 30). The effect of the device was evaluated using three key outcomes. First, mean total scores were assessed to track transitions between the four sleep-quality categories defined by the K-RCSQ. Second, the attainment of the minimal clinically important difference (MCID) in ISI and PSQI scores was evaluated. Lastly, ISI and PSQI scores were monitored to determine whether they reached remission thresholds during the trial.

## 3 Results: patient reported outcomes and clinical observations with biofeedback therapy

**Figure 3:**
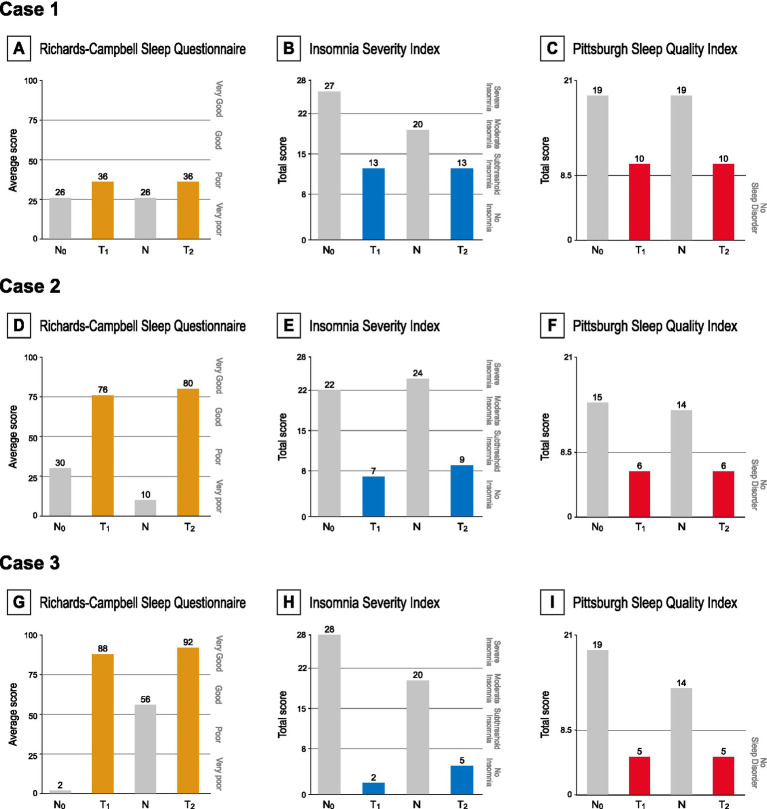
**(A,D,G)** RCSQ scores at each time point, categorized as very poor (0–25), poor (26–50), good (51–75), or very good (76–100). **(B,E,H)** ISI scores at each time point, interpreted as absence of insomnia (0–7), sub-threshold insomnia (8–14), moderate insomnia (15–21), or severe insomnia (22–28). A score of 15.5 is utilized as the cutoff threshold for diagnosing insomnia. **(G,F,I)** PSQI scores at each time point, with 8.5 used as the cutoff for insomnia.

Figure 3 illustrates K-RCSQ, ISI-K, and PSQI-K results plotted at each assessment point. The primary assessment, subjective sleep quality measured by the K-RCSQ, demonstrated clear on–off patterns across all three cases, with scores improving during intervention periods, declining during discontinuation, and improving again upon reintroduction of the intervention. Notably, Case 2 demonstrated a progression from “poor sleep” to “very good sleep,” while Case 3 exhibited a striking improvement from “very poor sleep” to “very good sleep” by the end of the trial.

Secondary assessments reflected similar improvements. ISI-K scores consistently declined across all three cases; by the end of both intervention periods, all patients had achieved the insomnia threshold defined in the validation study of ISI-K (ISI < 15.5 points) (29). Reductions exceeding the score of 6, determined as the MCID (31), were observed during both intervention phases in all three cases. Moreover, remission thresholds (ISI < 8 points) (32) were met by Cases 2 and 3 at the end of the first intervention period, and Case 3 achieved remission again after the shorter second intervention period. Similarly, PSQI-K scores demonstrated significant improvements across all three cases as Cases 2 and 3 achieved scores below the insomnia threshold at the end of both intervention periods. All patients exceeded the native version’s threshold for a clinically meaningful response (∆PSQI > 3 points) by the end of each intervention phase (33–35).

## 4 Discussion

This case series provides preliminary evidence supporting the feasibility and effectiveness of a smartphone-based biofeedback intervention for managing insomnia in subacute stroke patients unresponsive to pharmacological treatment. Insomnia is a complex multifactorial disorder arising from a web of interconnected physiological mechanisms, with autonomic dysfunction recognized as one contributing factor (36–38). Although the exact mechanisms remain debated, a similar process is likely responsible for insomnia in stroke patients. Studies involving Heart Rate Variability (HRV) analysis and plasma norepinephrine levels indicate that acute cerebrovascular events disrupt the autonomic nervous system (ANS), characterized by heightened sympathetic (SNS) activity and reduced parasympathetic (PNS) activity (39–41). In particular, right-sided insular cortex lesions have been strongly associated with heightened SNS outflow, reduced PNS activity, and lower HRV, suggesting a potential link between stroke-related autonomic dysfunction and insomnia (42–44).

Building on this understanding of ANS dysfunction in insomnia patients, the biofeedback application used in this study offers an innovative approach to addressing insomnia by potentially modulating autonomic function. It is hypothesized to restore autonomic balance through mechanisms distinct from conventional treatments, including increasing the magnitude of respiratory sinus arrhythmia (RSA) via controlled breathing and stimulating slowly adapting pulmonary stretch receptors through regulated lung inflation (45, 46). These processes may synergistically activate the vagus nerve, enhance parasympathetic activity, suppress sympathetic activity, and restore ANS homeostasis, which in turn could contribute to improving post-stroke insomnia (47–49). This proposed mechanism is partially supported by prior studies that have demonstrated efficacy of vagus nerve stimulation (VNS) in treating insomnia and autonomic dysfunction (50, 51). However, VNS’s invasive nature and reliance on external electrical stimuli limit its applicability. In contrast, this biofeedback application may offer a non-invasive alternative that replicates the benefits of VNS.

The results of this study further validate the proposed mechanism, demonstrating its effectiveness in patients previously unresponsive or minimally responsive to pharmacotherapy. Unlike traditional treatments, the biofeedback application is hypothesized to stimulate the vagus nerve and may help circumvent some of the challenges associated with impaired monoaminergic and GABAergic systems often seen in stroke patients (52–54). Improvements across primary and secondary assessments underscore its efficacy in addressing post-stroke insomnia. Notably, categorical improvements in K-RCSQ scores for Cases 2 and 3, including progression to higher sleep quality categories, highlight its potential to alleviate significant symptoms. Marked reductions in ISI scores, with all three cases exceeding the MCID and Cases 2 and 3 reaching remission thresholds, along with substantial PSQI improvements surpassing the MCID for all three patients, further support its therapeutic benefits. The on–off design of the study suggests a potential causal relationship, as improvements appeared to align consistently with the application’s use and declined during the washout period. However, as this was a proof-of-concept study involving a small number of participants, the strength of this inference is limited. Furthermore, because no control group was incorporated, the influence of uncontrolled confounding factors such as natural stroke recovery, concurrent medications, and placebo effects cannot be completely ruled out.

This study highlights several advantages of biofeedback interventions for insomnia in PSSD, with safety being the primary benefit. Unlike pharmacological therapies, which carry risks of adverse effects that may impair cognition and motor control in stroke recovery patients, the biofeedback approach primarily relies on internal physiological adjustments. By avoiding the introduction of external chemicals or stimulants, this method minimizes the risk of disrupting the delicate neurochemical and neuronal balance, thereby ensuring a superior safety profile. Notably, no common side effects typically associated with conventional pharmacotherapy treatments - such as dizziness, oversedation, or headaches (55)—were observed during this trial in all three cases, further supporting the intervention’s favorable safety profile. Additionally, the first case subject, who had previously experienced dizziness as an adverse effect of zolpidem tartrate, did not report similar or new symptoms during the intervention phase. Such finding indicates that the intervention is well-tolerated, even in individuals with heightened sensitivity to drug-related adverse effects.

Moreover, the intervention demonstrated a rapid onset of effects, with noticeable benefits observed during the first treatment window, likely reflecting its direct modulation of the ANS function. This rapid onset of effect is particularly significant, as quality sleep during the early stages of rehabilitation is closely linked to an improved quality of life (56, 57). The ability to achieve significant results within a short time frame sets this intervention apart from therapies such as CBT or dCBT, which require extended patient engagement and cognitive restructuring over weeks or months to yield comparable benefits. By addressing sleep disturbances promptly, the intervention used in this study has the potential to improve both short-term and long-term recovery trajectories for stroke patients (58).

The application’s straightforward design, requiring only a smartphone, eliminates the need for specialized equipment or trained personnel. This simplicity enhances its feasibility in clinical settings and its potential for home use. Additionally, caregivers found it effective in helping patients sleep without overnight delirium, significantly reducing their workload and the need for constant monitoring. Primary physicians also noted that the application required no supervision, allowing them to focus on other tasks, while the reduction in shift calls minimized overnight interventions. Other patients in the same ward also experienced improved sleep quality, as the absence of disruptions caused by case patients created a more conducive sleep environment. These findings demonstrate that the application benefits not only individual users but also caregivers, healthcare staff, and other patients in shared environments.

This study has several limitations that should be addressed in future research. First, the findings are based on a small sample size of three subacute stroke inpatients. To confirm the application’s efficacy, larger and more diverse patient populations, including outpatients and those with chronic PSSD, should be studied within the framework of RCTs. Additionally, this study only evaluated short-term intervention use. Future research should investigate the effects of long-term application use to determine if the benefits persist over extended periods. Lastly, the study did not incorporate objective sleep measures such as actigraphy or polysomnography.

This case report underscores the potential of a smartphone-based biofeedback application as an effective and safe intervention for managing insomnia in PSSD patients. Future research should focus on validating the application’s efficacy through RCT with placebo comparisons, determining the optimal duration of intervention, and evaluating the long-term sustainability of its therapeutic benefits.

## Data Availability

The raw data supporting the conclusions of this article will be made available by the authors without undue reservation.

## References

[1] Duss SB Seiler A Schmidt MH Pace M Adamantidis A Müri RM. The role of sleep in recovery following ischemic stroke: a review of human and animal data. Neurobiol Sleep Circadian Rhythms. (2017) 2:94–105. doi: 10.1016/j.nbscr.2016.11.003, PMID: 31236498 PMC6575180

[2] Fulk GD Boyne P Hauger M Ghosh R Romano S Thomas J. The impact of sleep disorders on functional recovery and participation following stroke: a systematic review and meta-analysis. Neurorehabil Neural Repair. (2020) 34:1050–61. doi: 10.1177/1545968320962501, PMID: 33153378

[3] Hermann DM Bassetti CL. Role of sleep-disordered breathing and sleep-wake disturbances for stroke and stroke recovery. Neurology. (2016) 87:1407–16. doi: 10.1212/wnl.0000000000003037, PMID: 27488603 PMC5047039

[4] Pasic Z Smajlovic D Dostovic Z Kojic B Selmanovic S. Incidence and types of sleep disorders in patients with stroke. Med Arch. (2011) 65:225. doi: 10.5455/medarh.2011.65.225-227, PMID: 21950229

[5] Baylan S Griffiths S Grant N Broomfield NM Evans JJ Gardani M. Incidence and prevalence of post-stroke insomnia: a systematic review and meta-analysis. Sleep Med Rev. (2020) 49:101222. doi: 10.1016/j.smrv.2019.101222, PMID: 31739180

[6] American Psychiatric Association. Diagnostic and statistical manual of mental disorders: DSM-5. Washington, DC: American Psychiatric Association (2013).

[7] American Psychiatric Association. Diagnostic and statistical manual of mental disorders: DSM-IV. Washington, DC: American Psychiatric Association (1994).

[8] Cai H Wang X-P Yang G-Y. Sleep disorders in stroke: an update on management. Aging Dis. (2021) 12:570–85. doi: 10.14336/ad.2020.0707, PMID: 33815883 PMC7990374

[9] Ellis JG Gehrman P Espie CA Riemann D Perlis ML. Acute insomnia: current conceptualizations and future directions. Sleep Med Rev. (2012) 16:5–14. doi: 10.1016/j.smrv.2011.02.002, PMID: 21596596

[10] Ford ME Groet E Daams JG Geurtsen GJ Van Bennekom CA Someren EJ. Non-pharmacological treatment for insomnia following acquired brain injury: a systematic review. Sleep Med Rev. (2020) 50:101255. doi: 10.1016/j.smrv.2019.101255, PMID: 31927422

[11] Riemann D Espie CA Altena E Arnardottir ES Baglioni C Bassetti CL. The European insomnia guideline: an update on the diagnosis and treatment of insomnia 2023. J Sleep Res. (2023) 32:e14035. doi: 10.1111/jsr.12625 38016484

[12] Sateia MJ Buysse DJ Krystal AD Neubauer DN Heald JL. Clinical practice guideline for the pharmacologic treatment of chronic insomnia in adults: an American academy of sleep medicine clinical practice guideline. J Clin Sleep Med. (2017) 13:307–49. doi: 10.5664/jcsm.6470, PMID: 27998379 PMC5263087

[13] Im KB Strader S Dyken ME. Management of sleep disorders in stroke. Curr Treat Options Neurol. (2010) 12:379–95. doi: 10.1007/s11940-010-0089-2, PMID: 20842596

[14] Devlin JW Mallow-Corbett S Riker RR. Adverse drug events associated with the use of analgesics, sedatives, and antipsychotics in the intensive care unit. Crit Care Med. (2010) 38:S231–43. doi: 10.1097/ccm.0b013e3181de125a, PMID: 20502176

[15] Ritterband LM Thorndike FP Ingersoll KS Lord HR Gonder-Frederick L Frederick C. Effect of a web-based cognitive behavior therapy for insomnia intervention with 1-year follow-up: a randomized clinical trial. JAMA Psychiatry. (2017) 74:68–75. doi: 10.1001/jamapsychiatry.2016.3249, PMID: 27902836

[16] Freeman D Sheaves B Goodwin GM Yu L-M Nickless A Harrison PJ. The effects of improving sleep on mental health (OASIS): a randomised controlled trial with mediation analysis. Lancet Psychiatry. (2017) 4:749–58. doi: 10.1016/s2215-0366(17)30328-0, PMID: 28888927 PMC5614772

[17] Soh HL Ho RC Ho CS Tam WW. Efficacy of digital cognitive behavioural therapy for insomnia: a meta-analysis of randomised controlled trials. Sleep Med. (2020) 75:315–25. doi: 10.1016/j.sleep.2020.08.020, PMID: 32950013

[18] Rossi S Hallett M Rossini PM Pascual-Leone A Group SoTC. Safety, ethical considerations, and application guidelines for the use of transcranial magnetic stimulation in clinical practice and research. Clin Neurophysiol. (2009) 120:2008–39. doi: 10.1016/j.clinph.2009.08.016, PMID: 19833552 PMC3260536

[19] Brunoni AR Amadera J Berbel B Volz MS Rizzerio BG Fregni F. A systematic review on reporting and assessment of adverse effects associated with transcranial direct current stimulation. Int J Neuropsychopharmacol. (2011) 14:1133–45. doi: 10.1017/s1461145710001690, PMID: 21320389

[20] Russo C Carneiro MIS Bolognini N Fregni F. Safety review of transcranial direct current stimulation in stroke. Neuromodulation. (2017) 20:215–22. doi: 10.1111/ner.12574, PMID: 28220641 PMC5389927

[21] Lehrer PM Gevirtz R. Heart rate variability biofeedback: how and why does it work? Front Psychol. (2014) 5:756. doi: 10.3389/fpsyg.2014.00756, PMID: 25101026 PMC4104929

[22] Li Q Steward CJ Cullen T Che K Zhou Y. Presleep heart-rate variability biofeedback improves mood and sleep quality in Chinese winter Olympic bobsleigh athletes. Int J Sports Physiol Perform. (2022) 17:1516–26. doi: 10.1123/ijspp.2022-0037, PMID: 35931415

[23] Park JJ Ha J Lee JE. Efficacy of a digital therapeutic intervention using self-regulating auditory biofeedback on sleep onset and maintenance in insomnia: a randomized crossover trial. Sleep Med. Under Review

[24] Kakar E Priester M Wessels P Slooter AJ Louter M van der Jagt M. Sleep assessment in critically ill adults: a systematic review and meta-analysis. J Crit Care. (2022) 71:154102. doi: 10.1016/j.jcrc.2022.154102 35849874

[25] Xiao M Huang G Feng L Luan X Wang Q Ren W. Impact of sleep quality on post-stroke anxiety in stroke patients. Brain Behav. (2020) 10:e01716. doi: 10.1002/brb3.1716, PMID: 33140545 PMC7749555

[26] Niu S Wu Q Ding S Wu L Wang L Shi Y. Comparison of three measures for insomnia in ischemic stroke patients: Pittsburgh sleep quality index, insomnia severity index, and Athens insomnia scale. Front Neurol. (2023) 14:1118322. doi: 10.3389/fneur.2023.1118322, PMID: 37712082 PMC10498538

[27] Cho YW Song ML Morin CM. Validation of a Korean version of the insomnia severity index. J Clin Neurol. (2014) 10:210. doi: 10.3988/jcn.2014.10.3.210, PMID: 25045373 PMC4101097

[28] Sohn SI Kim DH Lee MY Cho YW. The reliability and validity of the Korean version of the Pittsburgh sleep quality index. Sleep Breath. (2012) 16:803–12. doi: 10.1007/s11325-011-0579-9, PMID: 21901299

[29] Yang M Morin CM Schaefer K Wallenstein GV. Interpreting score differences in the insomnia severity index: using health-related outcomes to define the minimally important difference. Curr Med Res Opin. (2009) 25:2487–94. doi: 10.1185/03007990903167415, PMID: 19689221

[30] Bastien CH Vallières A Morin CM. Validation of the insomnia severity index as an outcome measure for insomnia research. Sleep Med. (2001) 2:297–307. doi: 10.1016/s1389-9457(00)00065-4, PMID: 11438246

[31] Shergis JL Ni X Jackson ML Zhang AL Guo X Li Y. A systematic review of acupuncture for sleep quality in people with insomnia. Complement Ther Med. (2016) 26:11–20. doi: 10.1016/j.ctim.2016.02.007, PMID: 27261976

[32] McDonnell LM Hogg L McDonnell L White P. Pulmonary rehabilitation and sleep quality: a before and after controlled study of patients with chronic obstructive pulmonary disease. NPJ Prim Care Respir Med. (2014) 24:1–5. doi: 10.1038/npjpcrm.2014.28 PMC4373390 25010602

[33] Eadie J van de Water AT Lonsdale C Tully MA van Mechelen W Boreham CA. Physiotherapy for sleep disturbance in people with chronic low back pain: results of a feasibility randomized controlled trial. Arch Phys Med Rehabil. (2013) 94:2083–92. doi: 10.1016/j.apmr.2013.04.017, PMID: 23643716

[34] Jarrin DC Ivers H Lamy M Chen IY Harvey AG Morin CM. Cardiovascular autonomic dysfunction in insomnia patients with objective short sleep duration. J Sleep Res. (2018) 27:e12663. doi: 10.1111/jsr.12663, PMID: 29493063 PMC5992004

[35] Bonnet MH Arand D. Heart rate variability in insomniacs and matched normal sleepers. Biopsychosoc Sci Med. (1998) 60:610–5. doi: 10.1097/00006842-199809000-00017, PMID: 9773766

[36] Kim H Jung HR Kim JB Kim D-J. Autonomic dysfunction in sleep disorders: from neurobiological basis to potential therapeutic approaches. J Clin Neurol (Seoul, Korea). (2022) 18:140. doi: 10.3988/jcn.2022.18.2.140, PMID: 35274834 PMC8926769

[37] De Raedt S De Vos A Keyser J. Autonomic dysfunction in acute ischemic stroke: an underexplored therapeutic area? J Neurol Sci. (2015) 348:24–34. doi: 10.1016/j.jns.2014.12.007, PMID: 25541326

[38] Korpelainen JT Sotaniemi KA Myllylä VV. Autonomic nervous system disorders in stroke. Clin Auton Res. (1999) 9:325–33. doi: 10.1007/bf02318379 10638806

[39] Dorrance AM Fink G. Effects of stroke on the autonomic nervous system. Compr Physiol. (2015) 5:1241–63. doi: 10.1002/cphy.c140016 26140717

[40] Nagai M Hoshide S Kario K. The insular cortex and cardiovascular system: a new insight into the brain-heart axis. J Am Soc Hypertens. (2010) 4:174–82. doi: 10.1016/j.jash.2010.05.001, PMID: 20655502

[41] Mo J Huang L Peng J Ocak U Zhang J Zhang JH. Autonomic disturbances in acute cerebrovascular disease. Neurosci Bull. (2019) 35:133–44. doi: 10.1007/s12264-018-0299-2, PMID: 30311072 PMC6357277

[42] Liu C-H Liu C-Z Zhang J Yuan Z Tang L-R Tie C-L. Reduced spontaneous neuronal activity in the insular cortex and thalamus in healthy adults with insomnia symptoms. Brain Res. (2016) 1648:317–24. doi: 10.1016/j.brainres.2016.07.024, PMID: 27425430

[43] Yasuma F Hayano J-i. Respiratory sinus arrhythmia: why does the heartbeat synchronize with respiratory rhythm? Chest. (2004) 125:683–90. doi: 10.1378/chest.125.2.683, PMID: 14769752

[44] Schelegle ES Green JF. An overview of the anatomy and physiology of slowly adapting pulmonary stretch receptors. Respir Physiol. (2001) 125:17–31. doi: 10.1016/s0034-5687(00)00202-4, PMID: 11240150

[45] Joseph CN Porta C Casucci G Casiraghi N Maffeis M Rossi M. Slow breathing improves arterial baroreflex sensitivity and decreases blood pressure in essential hypertension. Hypertension. (2005) 46:714–8. doi: 10.1161/01.hyp.0000179581.68566.7d, PMID: 16129818

[46] Jerath R Edry JW Barnes VA Jerath V. Physiology of long pranayamic breathing: neural respiratory elements may provide a mechanism that explains how slow deep breathing shifts the autonomic nervous system. Med Hypotheses. (2006) 67:566–71. doi: 10.1016/j.mehy.2006.02.042, PMID: 16624497

[47] Kubin L Alheid GF Zuperku EJ McCrimmon DR. Central pathways of pulmonary and lower airway vagal afferents. J Appl Physiol. (2006) 101:618–27. doi: 10.1152/japplphysiol.00252.2006, PMID: 16645192 PMC4503231

[48] George MS Ward HE Jr Ninan PT Pollack M Nahas Z Anderson B. A pilot study of vagus nerve stimulation (VNS) for treatment-resistant anxiety disorders. Brain Stimul. (2008) 1:112–21. doi: 10.1016/j.brs.2008.02.001, PMID: 20633378

[49] Kamel LY Xiong W Gott BM Kumar A Conway CR. Vagus nerve stimulation: an update on a novel treatment for treatment-resistant depression. J Neurol Sci. (2022) 434:120171. doi: 10.1016/j.jns.2022.120171, PMID: 35158102

[50] Hama S Murakami T Yamashita H Onoda K Yamawaki S Kurisu K. Neuroanatomic pathways associated with monoaminergic dysregulation after stroke. Int J Geriatr Psychiatry. (2017) 32:633–42. doi: 10.1002/gps.4503, PMID: 27251297

[51] Saji M Cohen M Blau AD Wessel TC Volpe BT. Transient forebrain ischemia induces delayed injury in the substantia nigra reticulata: degeneration of GABA neurons, compensatory expression of GAD mRNA. Brain Res. (1994) 643:234–44. doi: 10.1016/0006-8993(94)90030-2, PMID: 8032919

[52] Clarkson AN Huang BS MacIsaac SE Mody I Carmichael ST. Reducing excessive GABA-mediated tonic inhibition promotes functional recovery after stroke. Nature. (2010) 468:305–9. doi: 10.1038/nature09511, PMID: 21048709 PMC3058798

[53] Lenhart SE Buysse DJ. Treatment of insomnia in hospitalized patients. Ann Pharmacother. (2001) 35:1449–57. doi: 10.1345/1542-6270(2001)035<1449:toiihp>2.0.co;2 11724098

[54] Tang W-K Grace Lau C Mok V Ungvari GS Wong K-S. Insomnia and health-related quality of life in stroke. Top Stroke Rehabil. (2015) 22:201–7. doi: 10.1179/1074935714z.0000000026, PMID: 25908494

[55] Kim W-H Jung H-Y Choi H-Y Park C-H Kim E-S Lee S-J. The associations between insomnia and health-related quality of life in rehabilitation units at 1 month after stroke. J Psychosom Res. (2017) 96:10–4. doi: 10.1016/j.jpsychores.2017.02.008, PMID: 28545786

[56] Kim W-H Yoo Y-H Lim J-Y Kang S-G Jung H-Y Bae JN. Objective and subjective sleep problems and quality of life of rehabilitation in patients with mild to moderate stroke. Top Stroke Rehabil. (2020) 27:199–207. doi: 10.1080/10749357.2019.1673591, PMID: 31618116

[57] Kwan Y Yoon S Suh S Choi S. A randomized controlled trial comparing neurofeedback and cognitive-behavioral therapy for insomnia patients: pilot study. Appl Psychophysiol Biofeedback. (2022) 47:95–106. doi: 10.1007/s10484-022-09534-6, PMID: 35147813

[58] Kremer S Blue T. Biofeedback as an adjunct or alternative intervention to cognitive behavioral therapy for insomnia. Sleep Med Clin. (2023) 18:85–93. doi: 10.1016/j.jsmc.2022.10.003, PMID: 36764789

