# Supplementary material for: Smartphone-based non-invasive biofeedback therapy for post-stroke sleep disorders: short report
Source: Front Neurol. 2025 Jun 25;16:1601821. doi: 10.3389/fneur.2025.1601821 (PMC12241809; doi:10.3389/fneur.2025.1601821)
Supplement: Supplementary file 1 [file Supplementary_file_1.pdf]

frontiers

Supplementary Material

Minimum Hardware & Platform Requirements

- Pipeline code: BELL-001
- Platform: Web browser–based application (no installation required)
- Device Compatibility: Compatible with most modern Android and iOS smartphones released after 2017
- Operating System: Android 8.0+ or iOS 12+
- RAM: 2GB or higher
- CPU: Quad-core processor or better
- Browser: Latest version of Chrome, Safari, or equivalent modern browser
- Internet: Stable internet connection (Wi-Fi or 4G/LTE)
